# Reliability of threshold determination using portable muscle oxygenation monitors during exercise testing: a systematic review and meta-analysis

**DOI:** 10.1038/s41598-023-39651-z

**Published:** 2023-08-04

**Authors:** Carlos Sendra-Pérez, Jose Luis Sanchez-Jimenez, Joaquín Martín Marzano-Felisatti, Alberto Encarnación-Martínez, Rosario Salvador-Palmer, Jose I. Priego-Quesada

**Affiliations:** 1 Research Group in Sports Biomechanics (GIBD), Department of Physical Education and Sports, Faculty of Physical Activity and Sport Sciences, Universitat de València, C/Gascó Oliag, 3, 46010 Valencia, Spain; 2 Red Española de Investigación del Rendimiento Deportivo en Ciclismo y Mujer (REDICYM), Consejo Superior de Deportes (CSD), Facultad de Ciencias de la Actividad Física y del Deporte, Campus d’Ontinyent, Laboratorio Biomecánica, Avda. Conde de Torrefiel n° 22, 46870 Ontinyent, Spain; 3 Biophysics and Medical Physics Group, Department of Physiology, Universitat de València, Faculty of Medicine and Odontology, Avd. Blasco Ibañez 15, 46010 Valencia, Spain

**Keywords:** Biotechnology, Physiology

## Abstract

Over the last few years, portable Near-Infrared Spectroscopy (NIRS) technology has been suggested for determining metabolic/ventilator thresholds. This systematic review and meta-analysis aimed to assess the reliability of a portable muscle oxygenation monitor for determining thresholds during exercise testing. The proposed PICO question was: Is the exercise intensity of muscle oxygenation thresholds, using portable NIRS, reliable compared with lactate and ventilatory thresholds for exercise intensity determined in athletes? A search of Pubmed, Scopus and Web of Science was undertaken and the review was conducted following PRISMA guidelines. Fifteen articles were included. The domains which presented the highest biases were confounders (93% with moderate or high risk) and participant selection (100% with moderate or high risk). The intra-class correlation coefficient between exercise intensity of the first ventilatory or lactate threshold and the first muscle oxygenation threshold was 0.53 (obtained with data from only 3 studies), whereas the second threshold was 0.80. The present work shows that although a portable muscle oxygenation monitor has moderate to good reliability for determining the second ventilatory and lactate thresholds, further research is necessary to investigate the mathematical methods of detection, the capacity to detect the first threshold, the detection in multiple regions, and the effect of sex, performance level and adipose tissue in determining thresholds.

## Introduction

In many sports, various methods of exercise testing are performed for detecting metabolic/ventilatory thresholds. These zones or points are characterized by nonlinear increases of physiological outcomes (e.g., dot(V), oxygen volume (VO_2_), blood lactate, heart rate, etc.) so determining two physiological breakpoints that allow the three-phase model of intensities to be applied^1–3^. These data are important to trainers and athletes for assessing physical condition and programming intensities to optimize training and improving cardiovascular fitness and endurance^4,5^. Therefore, it is of great importance to have a reliable method for threshold detection^6^.

The ventilatory or metabolic threshold is usually determined by gas exchange or blood lactate data respectively, obtained during incremental tests^4,7^. Gas exchange is one of the most commonly used methods for assessing the evolution of gas exchange measurements (dot(V), VO_2_, carbon dioxide volume (VCO_2_) and minute ventilation (VE)) that allow detection of the respiratory compensation point (also referred to as ventilatory threshold (VT))^8^. For example, one method that is often used is the ventilatory method consists of determining the first and second ventilatory thresholds by detecting nonlinear increases in minute ventilation, the ventilatory equivalent for oxygen, the ventilatory equivalent for carbon dioxide, oxygen uptake, and carbon dioxide production^9^. Another widely used method is the blood lactate measurement^10^. In contemporary physiology, lactate is considered a major metabolic intermediate that has a wide-ranging impact on energy substrate utilization, cell signaling, and adaptation^11^. It is also important for the mitochondria since lactate is the end product of glycolysis and plays a role in connecting oxygen-independent and oxygen-dependent energy production, as a major energy source for mitochondrial respiration^4,11^. Hence, lactate enters the mitochondrial reticulum to support cell energy homeostasis by oxidative phosphorylation, and this process helps lactate disposal^11^. Threshold determination using blood lactate concentration can be obtained from values fixed (e.g., 2 or 4 mmol L^−1^)^12^ to mathematical models^13,14^.

However, both methods have associated limitations such as the economic cost of gas exchange, and the necessity to extract a drop of blood or its incapacity to measure continuously for lactate^15^, all of which makes it interesting to explore new methodologies. Moreover, it has been suggested that determining thresholds using muscle oxygen saturation (SmO_2_) could be a valid alternative to pulmonary gas exchange or blood lactate methods^16,17^.

Muscle oxygenation based on Near-Infrared Spectroscopy (NIRS) is a non-invasive technology that was described for the first time by Jöbsis in 1977, for monitoring in vivo cerebral oxygenation^18^. Nowadays, it is becoming very popular in the sports training field, thanks to the appearance of more affordable, easy to apply, and portable measuring devices^19,20^. Currently, NIRS technology is based on the modified Beer-Lambert’s law, which considers the dispersion of the nature of the tissues and their geometry^21,22^ (Eq. 1). NIRS technology detects the oxyhemoglobin ([O_2_Hb]) or deoxyhemoglobin ([HHb]) depending on light absorption, but in both cases, hemoglobin or myoglobin are referenced, since NIRS technology does not differentiate between chromophores (Eq. 2).

1 $$A=log\frac{I}{{I}_{O}}= \varepsilon \left[C\right]L*DPF+G$$

Modified Beer-Lambert’s law Eq. (1), where “A” is the absorption, “I” is the luminous intensity (lm sr^−1^), “$\upvarepsilon$” is the extinction coefficient for the light absorbing compound of interest, “[C]” is the concentration of the compound of interest (e.g. [Hb], [Mb] and/or [cyt_ox_]), “L” is the source-detector distance (mm), “DPF” the differential path length factor and “G” is the factor reflecting non-absorption.

2 $$Sm{O}_{2}=\frac{{O}_{2}Hb}{{O}_{2}Hb+HHb} \times 100$$

Equation for calculating muscle oxygen saturation (SmO_2_) by the oxyhemoglobin (O_2_Hb) and deoxyhemoglobin (HHb) measured.

NIRS technology in the sports field is being used to observe changes in the muscle metabolism of different muscles^19^. This has allowed us to measure local muscle performance during exercise, determining whether the muscles work optimally and if there is deoxygenation depending on exercise intensity^20,23,24^. Moreover, although several studies have suggested that portable NIRS technology can be used for determining muscle oxygenation thresholds^17,25,26^, and many studies have been published over the last few years, as far as the author knows, no systematic reviews and meta-analyses that validate the use of NIRS technology to detect thresholds have been undertaken.

Therefore, the aim of this systematic review and meta-analysis was to evaluate the reliability of determining the exercise intensity of the muscle oxygenation threshold (using the portable NIRS) compared with detection, using a gold standard method during laboratory and field tests.

## Methods

### Literature search methodology

This systematic review and meta-analysis was carried out following the Preferred Reporting Items for Systematic Reviews and Meta-Analyses (PRISMA) statement^27^. The proposed PICO (Population, Intervention, Comparison and Outcomes of an article) question was: Is the exercise intensity of muscle oxygenation thresholds, using portable NIRS, reliable compared with lactate and ventilatory thresholds for exercise intensity determined in athletes? Three databases (PubMed, Scopus and Web of Science) were electronically searched on the 15th of June of 2023 using the following terms: “NIRS” OR “Near Infrared Spectroscopy” OR “muscle oxygenation” OR “oximetry” AND with the terms and synonyms “threshold” OR “breakpoint” OR “inflection point”. Additionally, (AND) different terms such as “exercise” OR “sport” OR “physical activity” OR “running” OR “cycling” OR “swimming” were used. Every database employed its own term mapping. The results were screened to identify relevant studies, first by abstract and finally by full text. Full texts underwent a thorough screening process to determine their eligibility for inclusion in the review. Only those texts that fulfilled all the predetermined criteria were considered for inclusion.

The articles obtained were exported to Zotero (version 6.0.15, Corporation for Digital Scholarship, Vienna, USA) to eliminate duplicates, and the abstracts were uploaded to JBI SUMARI (The University of Adelaide, Adelaide, Australia) to carry out the first screening.

### Inclusion and exclusion criteria

The inclusion criteria established for the systematic review were as follows: (1) Only studies written in English, Spanish or Portuguese, (2) studies using a portable and commercial NIRS for muscle oxygenation threshold detection, (3) studies using a gold standard (gas exchange or blood lactate methods) in addition to muscle oxygenation for thresholds detection, (4) studies with a healthy population between 18 and 65 years of age, and (5) experimental and quasi-experimental studies.

### Study selection and data extraction

The first screening was performed by reviewing the abstracts of articles, after removing duplicates. Then, the selected articles were fully read to reach a decision. The entire process was carried out by two reviewers. When there was a disagreement on an abstract or article, it was subsequently discussed until a consensus was reached. For each study, the extracted data were: the authors and the year, the participants, a short description of the protocol, the thresholds calculated, the NIRS brand, the NIRS location, and the results. The data from each included article were extracted by two reviewers and confirmed by a third. Participants were categorized as elite, highly trained, trained and recreationally active following previous guidelines^28,29^.

### Risk of bias and quality of evidence assessment

The quality of the quasi-experimental studies included in the systematic review was assessed by two reviewers working independently using the ROBINS-I Scale. The ROBINS-I Scale evaluates risk of bias across 7 domains: confounding, selection of participants, classification of interventions, deviations from intended interventions, missing data, measurement of outcomes and the selection of the reported results^30^. For each domain, the risk of bias assessment was categorized: no information, critical, serious, moderate or low^30^. When there was a disagreement between the reviewers a third reviewer was consulted.

### Meta-analysis

A separate meta-analysis was performed to examine the reliability in determining intensity at each threshold using NIRS and the gold standard method (gas exchange and/or blood lactate). The intraclass correlation coefficient (ICC) and sample size were extracted for each study. For the studies that did not provide ICC values, the ICC value was calculated from obtaining the data from the datasets, tables and figures of the article, or on request from the authors. In the case of figures, data was extracted from scatter plots using the plot digitizer application^31^. If the data were not provided by the authors, the study was excluded from the analysis. ICC values were calculated based on a single rater-measurement, absolute-agreement, and 2-way random-effects model. For studies where it was possible to obtain more than one ICC value (e.g., because the intensity at the threshold was extracted using different automatic methods), these ICC values were averaged, using only one ICC value for each study to avoid statistical dependence^31,32^. ICC values were transformed to Fisher’s z scale and a random-effects model with Restricted Maximum Likelihood Estimation was used for the analysis^33^, assessing the type of gold standard compared (gas exchange or blood lactate) as a possible moderator. Q and *I*^2^ statistics were used for the homogeneity analysis. *I*^2^ values of around 25%, 50%, and 75% denoted low, moderate, and large heterogeneity, respectively. To assess the publication bias, funnel plot with Duval and Tweedie’s trim-and-fill method for imputing missing data and the Egger’s test were performed^34,35^. To facilitate the interpretation of the data, Fisher's z values were then converted back to ICC values after completing the meta-analyses^33^. The ICC and associated 95% confidence intervals were interpreted as: poor (0.00–0.25), fair (0.26–0.50), moderate (0.51–0.75) and good (0.76–1.00)^36^. Statistical significance was established at p < 0.05. A meta-analysis was performed with the “metafor*”* package (version 4.2-0)^37^ in RSTUDIO (version 2023.06.0)^38^.

## Results

### Study selection

**Figure 1:**
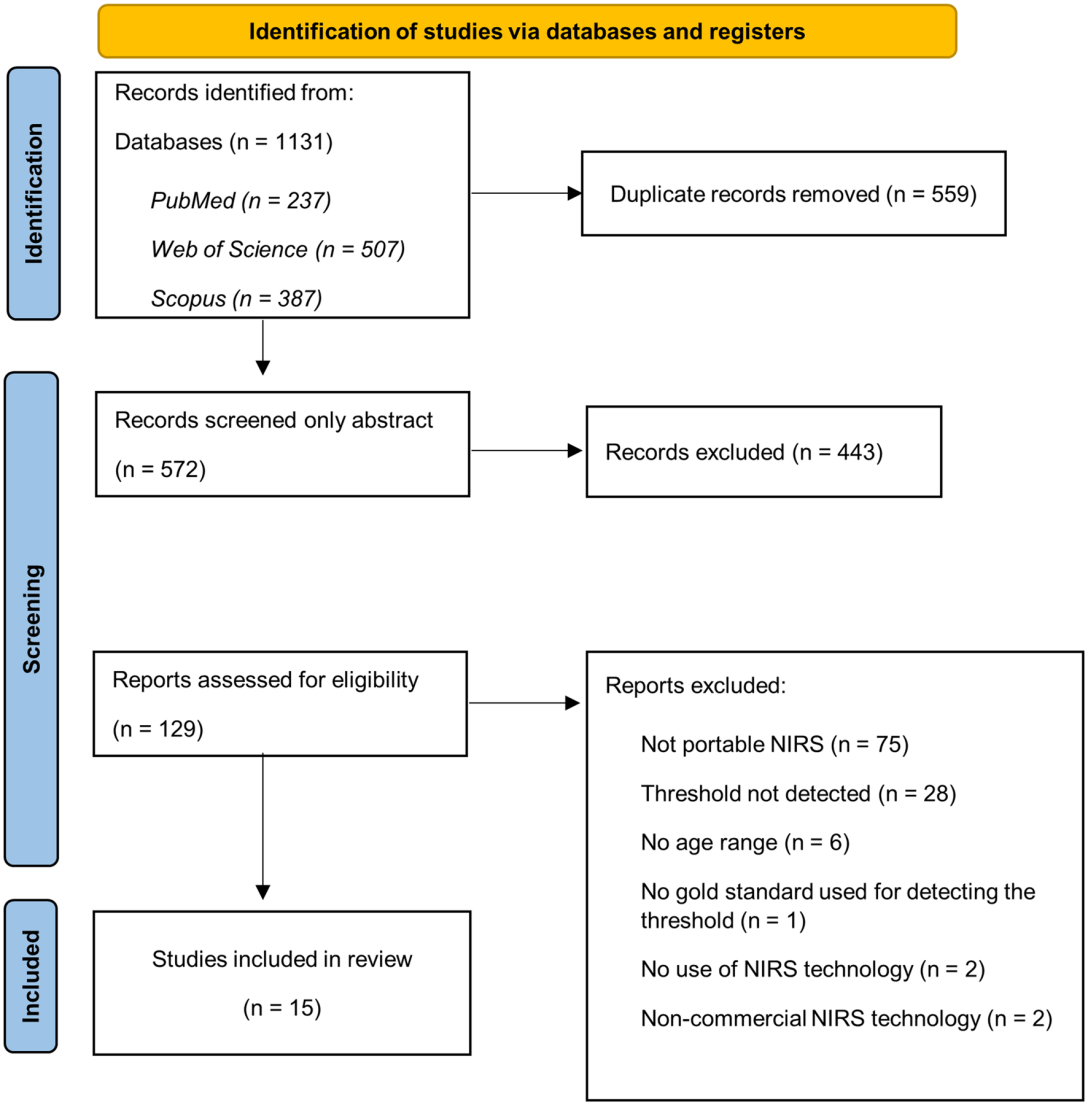
Study selection from the systematic review and meta-analysis (PRISMA).

A total of 1,131 articles from databases of PubMed (237), Web of Science (507), and Scopus (387) were included, and 559 articles remained after removing duplicates. Finally, after selecting studies by their abstracts, 129 full articles were reviewed, of which 15 were included in the systematic review (Fig. 1).

### Participants characteristics

The systematic review included a sample of 344 participants (216 males and 128 females). Among these participants 33 were elite athletes, 208 highly trained athletes, 31 trained athletes and 72 recreationally active athletes. Moreover, athletes from various sports were included (soccer, cycling, running, triathlon and rowing) with laboratory protocols, since there are currently no studies carried out in field tests. The study characteristics and the main findings are summarized in Table 1.

**Table 1:** Summary of selected studies.

Study (year)	Participants^a^	Protocol	Thresholds	NIRS device	NIRS location	Results/conclusions
Batterson et al.^44^	N = 10 (M) *Elite* *Soccer players*	**GXT** 3-min work + 30 s rest 9.0 km·h^−1^ W-UP ↑ 1.8 km·h^−1^ every 180 s *Treadmill	LT_2,_ LT_1_	Moxy	VL, GC, BF	MOT_1_ and MOT_2_ showed similar that LT_1_ and LT_2_ in all muscles analyzed. This show that SmO_2_ is useful for coaches
Borges and Driller^45^	N = 7 (M); 7 (F) *Highly trained athletes*	**GXT** 3-min W-UP (4.8 km·h^−1^) 3-min (9.3–11.7 km·h^−1^) ↑ 0.3–1.1 km·h^−1^ every 180 s *Treadmill	LT_2_	BSX Insight	GC	MOT_2_ showed a high correlation. The wearable lactate threshold sensor could be implemented by coaches and athletes
Cayot et al.^46^	N = 9 (M); 5 (F) *Recreationally*	**2 × GXT** *(separated by 7–10 days)* 5-min W-UP (25 W) ↑ 25 W every 180 s *Cycle ergometer	LT_2_	Moxy	VL	MOT_2_ detection was moderately correlated with the LT_2_ and the heart rate. The results do not support the use of two different mathematical methods for MOT_2_ determination
Contreras-Briceño et al.^39^	N = 8 (M); 7 (F) *Highly trained triathletes*	**GXT** 2-min rest 3-min W-UP (100 W) ↑ 20 W every 80 s *Bike on a cycle ergometer	VT_2_	Moxy	7th IC	A good-to-excellent correlation was obtained between MOT_2_ and VT_2_ for each variable of all analyses in the 7th IC, muscle
Driller et al.^47^	N = 10 (M); 5 (F) *Highly Trained Cyclists*	**GXT** 3-min 80–120 W ↑ 20 W every 180 s *Bicycle on an ergometer	LT_2_	BSX Insight	GC	LT_2_ determination through MOT_2_ showed an excellent correlation during cycling. These results were shown in all methods for LT_2_ detection
Farzam et al.^48^	N = 15 (M); 3 (F) *Recreationally*	**GXT** 4-min 30 W ↑30 W every 240 s *Cycle ergometer	LT_2_	Humon Hex, MetaOX*	RF	MOT_2_ determination showed good agreements with LT_2_ NIRS portable and NIRS non-portable showed a good correlation during the exercise. A low-cost, wireless, wearable NIRS is a good predictor of the threshold
Feldmann et al.^16^	N = 6 (M); 4 (F) *Recreationally cyclists and runners*	**GXT** Run test: 5-min W-UP (3.0–3.5 km·h^−1^) ↑0.5 km·h^−1^ every 30 s Cycling test: 5-min W-UP (50–100W) ↑25W every 25 s *Treadmill *Cycle ergometer	VT_1,_ VT_2_	Moxy	VL	NIRS technology is suitable for determining VT1 and VT2. Additionally, SmO_2min_ is a good indicator of cardiorespiratory fitness, as it correlated with VO_2peak_. Furthermore, no matter in which lateral vastus (right or left) the NIRS device was placed and the modality (cycling or running) it detected the MOT correctly
McMorries et al.^49^	N = 7 (M); 14 (F) *Trained* *Triathletes*	**GXT** ↑ 12–18 s per km every 180 s *Treadmill	LT_2_	BSX Insight	GC	MOT_2_ showed similar values to LT_2_, when the thresholds were compareted using the heart rate
Raleigh et al.^41^	N = 31 (M) *Highly trained cyclist/triathletes*	**GXT** 15-min W-UP (120 W) 3-min (100 W) ↑ 25 W every 180 s *Cycle Ergometer	LT_2_, VT_2_	Moxy	VL	MOT_2_, LT_2_ and VT_2_ were not different, but a poor correlation was obtained between them A good correlation was identified between VT_1_ and LT_1_
Rodrigo-Carranza et al.^25^	N = 5 (M); 5 (F) *Highly trained runners*	**GXT** 5-min W-Up (9 km·h^−1^) ↑ 1 km·h^−1^ every 60 s *Treadmill	VT_2_	Humon Hex	VL	VT_2_ and MOT_2_ were positively correlated during running. Thus, the device presented a good predictor of the second threshold
Osmani et al.^40^	N = 16 (M); 5 (F) *Recreationally*	**GXT** 3-min work + 30 s rest 8.0 km h^−1^ W-UP ↑ 1.2 km h^−1^ every 180 s *Treadmill	VT_2_	Humon Hex	VL	SmO_2_ data alone were not enough to determine the VT_2_ Also, SmO_2_ values of this device (Humon) do not correlate with other variables (blood lactate, RPE, HR and running power)
Salas-Montoro et al.^17^	N = 32 (M); 58 (F) *23 Elite* *67 Highly trained* *Cyclists*	**GXT** 5-min W-Up (15–20% of FTP) ↑ 25 W every 60 s *Cycle ergometer	LT_2_	Humon Hex	RF	LT_2_ was excellently correlated with MOT_2_ when compared using power output, percentage of maximal aerobic power, heart rate and percentage of maximum heart rate to MOT. The reliability of methods showed very good or excellent values in all cases (0.74–0.99) NIRS portable device can be an interesting tool for threshold detection for coaches without performing an on-site lactate test
Turnes et al.^26^	N = 13 (M) *Highly trained rowers*	**(1) GXT** 3-min (130 W) ↑30 W every 180 s//R 30″ **(2)** 10-min W-Up + 5-rest 2000 m test *Rowing ergometer	LT_2_	Portamon	VL	LT_2_ was moderately related to MOT_2_ during the rowing incremental test. However, the SmO_2_ in the VL presented a large variability between participants
Van der Zwaard et al.^42^	N = 30 (M); 10 (F) *9 Recreationally* *10 Trained* *21 Highly trained* *Cyclist and endurance trained*	**GXT** 3-min 1.5 W·kg^−1^ (85–145 W) ↑ 0.5 W·kg^−1^ (30–50 W) every 180 s *Cycle ergometer	VT_1_, VT_2_	Portamon	VL	VT_1_ and VT_2_ were moderately related to MOT. The relationship increased in trained cyclists (0.68–0.84) compared with recreationally trained males (0.48–0.50) VT differed across sexes and training status, whereas MOT differed only across sexes
Yogev et al.^43^	N = 17 (M); 5 (F) *Highly trained* *Cyclist*	**GXT** 6-min W-Up (110–140 W) 4-min (70–100 W) ↑1 W every 2 s *Stationary bicycle trainer	VT_2_	Moxy	LD, VL	VT_2_ and MOT_2_ showed a moderate relationship in both muscles The athletes and trainers could use portable NIRS to detect MOT

W: watts; M: male; F: female; GXT: graded exercise test; W-UP: warm up; R: recovery; LT_1_: first lactate threshold; LT_2_: second lactate threshold; VT_1_: first ventilatory threshold; VT_2_: second ventilatory threshold; BF: biceps femoris; LD: lateral deltoid; IC: intercostal; RF: rectus femoris; VL: vastus lateralis; GC: gastrocnemius; RCP: respiratory compensation point; MOT_1_: first muscle oxygenation threshold; MOT_2_: second muscle oxygenation threshold.

^a^Data are expressed as mean ± standard deviation.

*Non-portable NIRS.

### Methods used for determining muscle oxygenation threshold

The studies selected had determined both muscle oxygenation threshold (MOT) (first and second) using different methods (Table 2). Most of the studies used the regression double linear representing 42% and wearable lactate threshold (WLT) was used in 25% of the studies included in the systematic review. Together, these two methods represented 67% of the studies included in the systematic review. However, visual identification was also used in two studies (17%).

**Table 2:** Methods for determining the muscle oxygenation threshold in the studies selected.

Methods for determining the MOT	N	(%)	Threshold	Studies
Regression double linear	7	46	MOT_1_ & MOT_2_	^16,26,39,41–44^^a^
Wearable lactate threshold (WLT)	3	20	MOT_2_	^45,47,49^
Visual identification *(decrease of more than 15%)*	3	20	MOT_2_	^17,25,40^^b^
Application Humon Beta	2	7	MOT_2_	^48^
D-max or modified D-max	1	7	MOT_2_	^46^

MOT_1_: first muscle oxygenation threshold, MOT_2_: second muscle oxygenation threshold.

^a^Also visually checked.

^b^Inflection point at SmO_2_ values at the same point as the VT_2_.

### Risk of bias evaluation

**Figure 2:**
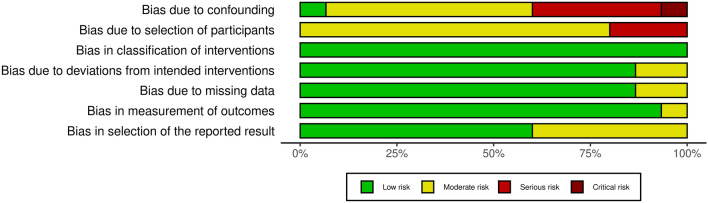
Risk of bias summary. Created with ‘robvis’ application^54^.

**Figure 3:**
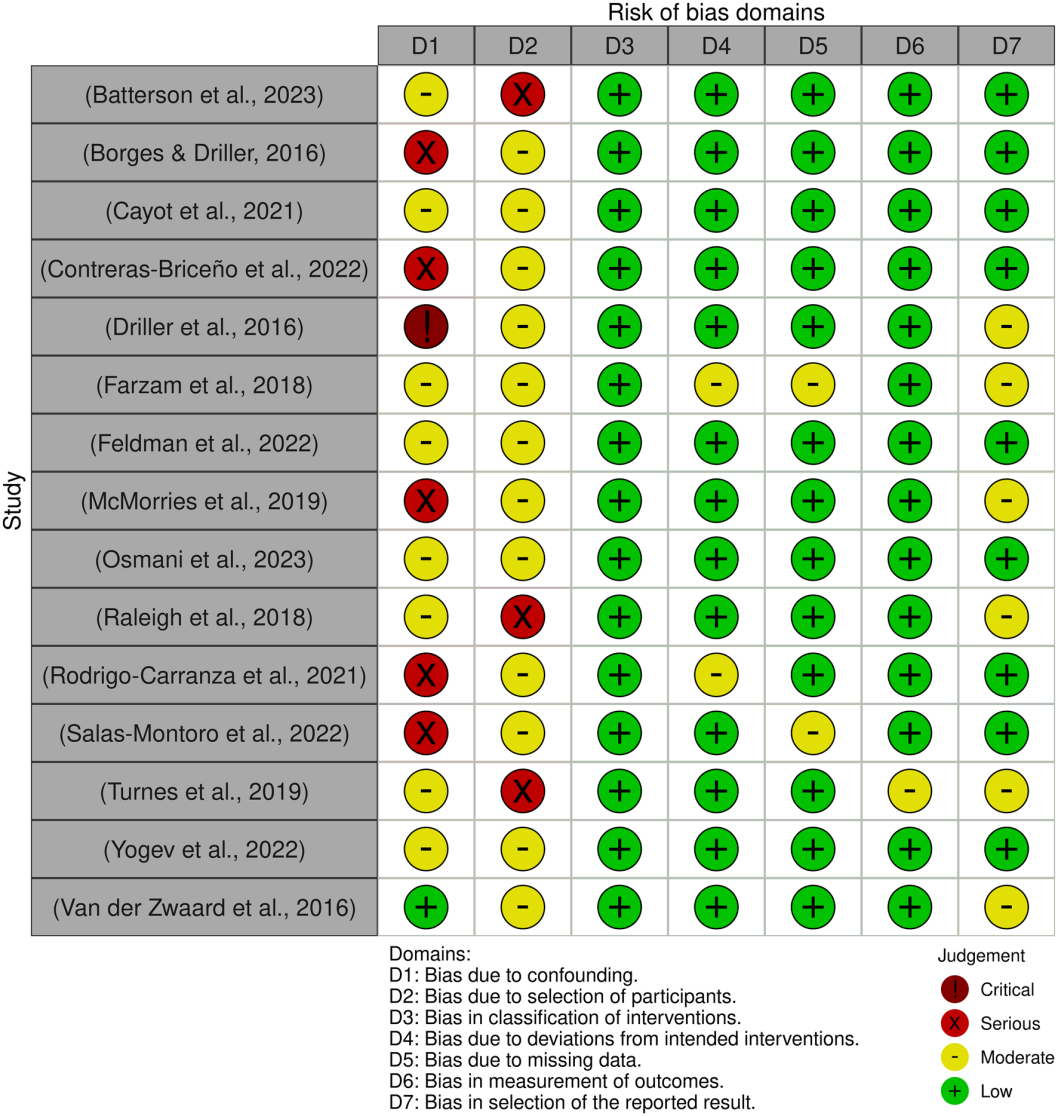
The risk of bias for each study. Created with ‘robvis’ application^54^.

The domains which presented the highest bias were due to confounding (7% with critical risk, 33% with serious risk and 53% with moderate risk), due to the selection of the participants (20% with serious risk and 80% with moderate risk), and due to the selection of the reported results (40% with moderate risk) (Figs. 2 and 3). For the other domains, most of the studies presented a low risk of bias (> 85%).

### Meta-analyses

Of the 15 articles included in this review, the ICCs of 13 of them were obtained from the meta-analysis (Table 3). Of these 13 articles, the ICC was provided in the article itself in 3, was calculated from the data obtained in a dataset, table or figure in 8, and in 2 the ICC was provided directly by the authors (Table 3).

**Table 3:** The intraclass correlations (ICC) for the exercise intensity of muscle oxygenation threshold and the gold standard.

Study	MOT method	Gold standard method	ICC source	*ICC*
Batterson et al.^44^	Segmented linear regression model	LT_1_ and LT_2_ was determined using a mDmax	Provided by the authors	LT_1_ right VL: 0.38 LT_1_ left VL: 0.08 **LT**_**1**_ ***ICC***_***mean***_**: 0.23** LT_2_ right VL: 0.54 LT_2_ right VL: 0.60 **LT**_2_ ***ICC***_***mean***_**: 0.57**
Borges and Driller^45^	Wearable lactate threshold sensor (WLT)	LT_2_ was determined using the following methods: LSF, Dmax, mDmax, 4 mmol·L^−1^ and an increase greater than 1 mmol·L^−1^	Article	*LSF: 0.91* *Dmax: 0.8* *mDmax: 0.89* *4mmoL: 0.98* *1mmoL: 0.92* ***ICC***_***mean***_**: *0.90***
Contreras-Briceño et al.^39^	Segmented linear regression model	VT_2_ was determined with the visual method by two blinded researchers	Calculated from data obtained from the Fig. 4 of the article	***ICC: 0.97***
Cayot et al.^46^	Dmax and modified Dmax	LT_2_ was determined using a Dmax and mDmax	Authors did not provide the dataset after requestion	–
Driller et al.^47^	Wearable lactate threshold sensor (WLT)	LT_2_ was determined using: TradLT, Dmax, mDmax and OBLA	Calculated from data obtained from the Fig. 2 of the article	*TradLT: 0.96* *Dmax: 0.88* *mDmax: 0.97* *OBLA: 0.96* ***ICC***_***mean:***_*** 0.94***
Farzam et al.^48^	Application Humon Beta	LT_2_ was determined using the value of 4 mmol·L^−1^ lactate	Authors did not provide the dataset after requestion	–
Feldmann et al.^16^	Segmented linear regression model	VT_1_ and VT_2_ were detected with a segmented regression analysis	Provided by the authors	*LT*_*1*_* running: 0.49* *LT*_*1*_ *cycling: 0.65* ***ICC***_***mean***_: ***0.57*** *LT*_*2*_* running: 0.92* *LT*_*2*_ *cycling: 0.92* ***ICC***_***mean***_: ***0.92***
McMorries et al.^49^	Wearable lactate threshold sensor (WLT)	LT_2_ was determined using the value of 4 mmol·L^−1^ lactate and an increase greater than 1 mmol·L^−1^	Calculated from data obtained from the Figure 6 of the article	***ICC: 0.29***
Osmani et al.^40^	Visual identification	VT_2_ was determined observing an inflection point	Calculated from data obtained from the Tables 1 and 2 of the article	***ICC: 0.23***
Raleigh et al.^41^	Segmented linear regression model	VT_2_ and LT_2_ were detected with a segmented regression analysis. The intersection of two linear segments was defined as the threshold	Article	**LT**_**2**_**: 0.54** **VT**_**2**_**: 0.36**
Rodrigo-Carranza et al.^25^	Visual identification	VT_2_ was identified by the nonlinear increase	Calculated from data obtained from the Table 1 of the article	***ICC: 0.84***
Salas-Montoro et al.^17^	Visual identification	LT_2_ was determined in an increase of at least 2 mmol·L^−1^ above baseline measurements	Article	***ICC: 0.91***
Turnes et al.^26^	Regression double linear and a visual identification	LT was determined by linear interpolation given a fixed concentration of 3.5 mmol·L^−1^	Calculated from data obtained from the Table 2 of the article	***ICC: 0.65***
Yogev et al.^43^	Regression double linear	Regression double linear was used to detect the threshold with WKO5. This is similar to the V-slope method	Calculated from dataset provided by the authors	VT_2_ VL: 0.73 VT_2_ LD: 0.79 ***ICC***_***mean:***_*** 0.76***
Van der Zwaard et al.^42^	Intercept of two congregating regression lines	VT detection method was the same as in MOT detection	Calculated from the dataset (supporting files) of the study	***ICC VT***_***1***_***: 0.56*** ***ICC VT***_***2***_***: 0.38***

ICC values used for the meta-analysis are in bold letters.

MOT_1_: first muscle oxygenation threshold; MOT_2_: second muscle oxygenation threshold; LT_1_: first lactate threshold; LT_2_: second lactate threshold; VT_1_: first ventilatory threshold; VT_2_: second ventilatory threshold; mDmax: modified Dmax; VL: vastus lateralis; LD: lateral deltoid.

Values used for the meta-analyses are in bold/italic.

**Figure 4:**
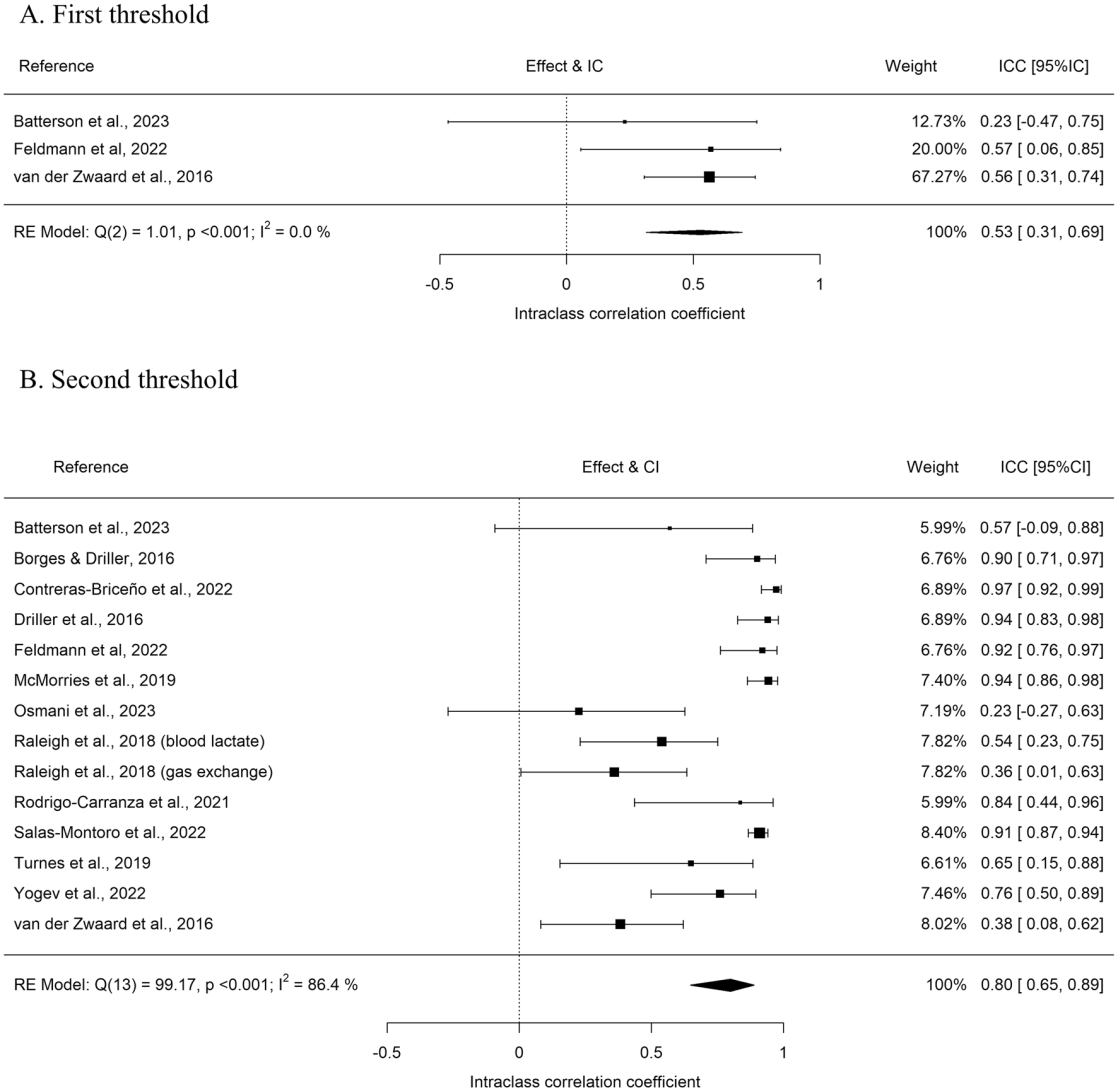
Forest plots of the meta-analysis was performed for the intraclass correlation (ICC) of the exercise intensity obtained at the first (**A**) and second (**B**) threshold determination using NIRS and the gold standard (gas exchange or blood lactate).

A test of moderators was not performed for the first threshold due to the low number of studies (n = 3, Table 3). The Q test was not significant (Q(df = 2) = 1.01, p-val = 0.60) and the* I*^2^ was 0%, showing a low heterogeneity. The Trim-and-fill method estimated 0 missing studies and Egger’s test was not significant (p = 0.46). The ICC of the first threshold was moderate (ICC = 0.53) but with a wide 95%CI[0.31, 0.69] (Fig. 4A).

For the second threshold, no effect of moderators was observed (p = 0.94) at first. Therefore, a meta-analysis was performed without differentiating between the ICC obtained compared with lactate or gas exchange. The Q test was not significant (Q(df = 13) = 99.17, p < 0.001) and the* I*^2^ was of 86%, showing a large heterogeneity. The Trim-and-fill method estimated 0 missing studies and Egger’s test was not significant (p = 0.54). The ICC of the second threshold was good (ICC = 0.80, 95%CI[0.65, 0.89] (Fig. 4B).

## Discussion

The aim of this systematic review and meta-analysis was to evaluate the reliability of determining exercise intensity using the muscle oxygenation threshold (with the portable NIRS) compared with a gold standard detection method during laboratory tests. The results of the review show that the methods mostly used to determine muscle oxygenation thresholds were regression double linear (46%), WLT (20%), and visual identification (20%). The meta-analysis revealed that of the 13 studies where ICC was obtained, only 3 studies assessed the first threshold, the mean ICC of 0.53 being observed between the exercise intensity obtained at the first muscle oxygenation threshold (MOT_1_) and first lactate threshold (LT_1_) or first ventilatory threshold (VT_1_). The mean ICC between second muscle oxygenation threshold (MOT_2_) and second lactate threshold (LT_2_) or second ventilatory threshold (VT_2_) was 0.80.

Our meta-analyses were focused on showing whether the exercise intensity where the first and second thresholds were detected using the portable NIRS was more reliable than the gold standards methods (gas exchange and blood lactate). Table 1 shows how the relationship between MOT and VT was analyzed in 7 studies^16,25,39–43^ and in 9 studies for LT^17,26,41,44–49^.

The studies of Feldmann et al.^16^ and Van der Zwaard et al.^42^ compared the VT_1_ and LT_1_ with MOT_1_ in cycling and found ICC values (ICC = 0.56–0.65). These results are in line with other studies that determined thresholds with non-portable NIRS in cycling^50^. Moreover, a fair ICC in running was shown (ICC = 0.23–0.49)^16,44^. 28/07/2023 17:06:00 A lower number of studies assessed the first threshold compared with the second one (3 vs. 12 studies), maybe due to the difficulty of determining the MOT_1,_ since the slope changes very slightly and the ICC value is not as good as the second threshold^42^.

The second threshold was determined using the blood lactate concentration and muscle oxygenation in different sports such as cycling^16,17,46–48^, running^44,45,49^ and rowing^26^. ICC values showed a certain disparity and were fair, moderate or good (ICC = 0.29–0.90) in studies of running, although cycling studies showed a good ICC (ICC = 0.91–0.94). However, the ICC value of two studies were not obtained^46,48^. The remaining studies also compared gas exchange with muscle oxygenation in the second threshold in cycling^16,39,42,43^ and running^16,40^. The results of the different studies suggest that the relationship between both methods in threshold determination is affected by the region assessed by the NIRS device, as good values (ICC = 0.92–0.97) were observed on assessing the intercostalis during cycling^39^. Moreover, the vastus lateralis presented moderate or good ICC in different investigations^25,42^, so the test or determination method chosen may also be critical.

Different methods were developed to determine the thresholds in blood lactate concentration and gas exchange, which are commonly combined by users to find the most optimal inflection point^51^. Despite recent research into the application of NIRS technology for the purpose of obtaining thresholds, there is a lack of research on its methods of determination. The articles included in this systematic review use different methods for determining thresholds: BSX Insight (20%, N = 3)^45,47,49^, double linear regression (46%, N = 7)^16,26,39,41–44^, visual method^17,25,40^, Dmax or modified Dmax^46^ and applications of devices Humon Beta^48^.

BSX Insight, which determines the threshold by making a comparison with blood lactate concentration, presented good values of ICC, although this used a patented method to determine MOT based on the inflection point of SmO_2_ during incremental testing^45^. However, as this system is commercial and patented, specific details of the algorithm used for said detection are unknown. Another important method is visual, which could be the most accurate for detecting the thresholds^17^ but with associated human error, or complementary to the previous one as was performed by Turnes et al.^26^ We recommend that future studies explore different methods to analyze thresholds using NIRS technology, to provide evidence on which are optimal, if several should be combined, or if some are more suitable for certain populations or sports.

The muscles analyzed with NIRS portable had previously been studied by Perrey & Ferrari^19^, who showed that SmO_2_ was determined among different muscles (vastus lateralis, gastrocnemius medialis, intercostal, triceps brachii) and many sports (swimming, strength, skiing, speed skating, sailing, running, rugby, climbing, handball, cycling, kayak, judo, rowing, football, alpine skiing). Vastus lateralis was the muscle most assessed^16,25,26,40–44,46^, although other muscles such as gastrocnemius^44,45,47,49^, rectus femoris^17^, biceps femoris^44^, lateral deltoid^43^ or intercostal^39^ were also evaluated. Moreover, the muscles analyzed in each study depend on the sports performed in the testing, the main muscles involved in that activity being selected. For example, in cycling the muscle most assessed was the vastus lateralis as it is the main muscle contributing to power output production. However, some studies explored other regions during cycling which could affect the determination of the threshold^17,47^, although the rectus femoris is also a power output producer in this area where there could be a higher proportion of adipose tissue^52^ or because its neuromuscular activation is not affected by the increase in workload during the test (e.g., gastrocnemius)^53^.

The systematic review also focused on exercise testing to determine whether the thresholds in the muscles (local thresholds) were analyzed or whether they are major exercise muscle. The articles included in this systematic review analyzed 1 or 3 muscles at most at the same time. Moreover, most of these studies were focused on correlating the main muscles of exercise with blood lactate concentration or gas exchange, and it is important to take into account that lactate and gas exchange determine systemic changes, while NIRS technology can be used for determining a more local response. For this reason, further studies that analyze different muscles simultaneously would be interesting in order to understand what is happening in each muscle during exercise testing, and how some may be more related to systemic changes while others have more specific alterations.

It is important to consider that the present meta-analysis is limited to only one measure of reliability (ICC), and more statistics are desirable (e.g., bias between methods) to improve the interpretation and application of the present results. Bias was not included due to the low number of studies that reported this data, and the different units used (W, km·h^−1^or percentage) also posed a challenge. This point should be regarded as a limitation of the present work, and future meta-analysis with a higher number of studies should incorporate more reliable statistics. Some of the articles included in this review demonstrate mean bias between MOT_2_ and LT_2_ or VT_2_ ranging from 0.01 and 0.4 km·h^−1^^25,44,45,49^, between 3.9 and 15.4 W^39,41^, 0.05 W·kg^−1^^17^ and 10.7% of the power output^26^. However, Batterson et al.^44^ showed a higher mean bias for MOT and LT_1_ (1.1–1.2 km·h^−1^), and Driller et al.^47^ also demonstrated how the method of determination could affect the bias, with the lowest being for the Dmax method (17 W) and the highest for the OBLA method (37 W). Finally, the study of Feldmann et al.^16^ stated that in terms of power or speed, the bias represents one performance step (for this particular study, it was 25 W for cycling and 0.5 km·h^−1^ for running).

Although the studies included present low risk of bias in most of the domains assessed, the analysis performed suggests that two domains presented a considerable risk of bias: confounders and the selection of the participants. The main issues related to the confounding domain were the studies that did not consider the effect of the training level of participants, prior activity or sex in their results. In some cases, only the value of correlation or intraclass correlation coefficient without the confidence interval appear in the reported results. However, the majority of studies had a missing data count bias and bias in measurement outcomes. Future studies should take into account these aspects, so as to control them as much as possible, to improve their quality and reduce their biases. Moreover, these aspects are possible sources of the high heterogeneity found in the meta-analysis.

The main limitation of the present work is the small number of studies included in the meta-analysis (N = 13). In future, a higher number of studies incorporated into the current analysis could corroborate the results obtained. Moreover, there was a high heterogeneity between the different studies included. Regarding the methodology, the regions or the sample assessed, with participants ranging from national and international level competitors^17^ to recreational ones^42^, could affect the results of the metanalysis.

Considering all the analyses carried out, we think that the following lines of research should be prioritized in this area: exploring which are the most appropriate mathematical detection methods depending on the sports or populations for NIRS, investigating whether it is possible to detect the first threshold, analyzing multiple regions at the same time to find out which ones are most related to systemic thresholds and which have a more specific behavior of the muscle itself, and understanding the differences in the detection of thresholds depending on sex, performance level, amount of adipose tissue or the changing of muscle length during exercise.

## Conclusion

The present systematic review and meta-analysis shows that, although using a portable muscle oxygenation monitor has moderate to good reliability for determining the second threshold, further research is necessary to investigate the mathematical methods of detection, the capacity to detect the first threshold, detection in multiple regions, and the effect of sex, performance level and adipose tissue on threshold determination.

## Data Availability

The datasets used and/or analysed during the current study available from the corresponding author on reasonable request.
